# Lipoxin A4 Preconditioning Attenuates Intestinal Ischemia Reperfusion Injury through Keap1/Nrf2 Pathway in a Lipoxin A4 Receptor Independent Manner

**DOI:** 10.1155/2016/9303606

**Published:** 2016-06-07

**Authors:** Xue Han, Weifeng Yao, Zipeng Liu, Haobo Li, Zhong-jun Zhang, Ziqing Hei, Zhengyuan Xia

**Affiliations:** ^1^Department of Anesthesiology, Sun Yat-sen Memorial Hospital, Sun Yat-sen University, Guangzhou 510000, China; ^2^Department of Anesthesiology, The Third Affiliated Hospital of Sun Yat-sen University, Guangzhou 510000, China; ^3^Department of Anaesthesiology, The University of Hong Kong, Pokfulam 999077, Hong Kong; ^4^Department of Anesthesiology, The Second Affiliated Hospital of Jinan University, Shenzhen 51000, China; ^5^Department of Anesthesiology, Affiliated Hospital of Guangdong Medical University, Zhanjiang 524001, China

## Abstract

Oxidative stress plays a critical role in the pathogenesis of intestinal ischemia reperfusion (IIR) injury. Enhancement in endogenous Lipoxin A4 (LXA4), a potent antioxidant and mediator, is associated with attenuation of IIR. However, the effects of LXA4 on IIR injury and the potential mechanisms are unknown. In a rat IIR (ischemia 45 minutes and subsequent reperfusion 6 hours) model, IIR caused intestinal injury, evidenced by increased serum diamine oxidase, D-lactic acid, intestinal-type fatty acid-binding protein, and the oxidative stress marker 15-F2t-Isoprostane. LXA4 treatment significantly attenuated IIR injury by reducing mucosal 15-F2t-Isoprostane and elevating endogenous antioxidant superoxide dismutase activity, accompanied with Keap1/Nrf2 pathway activation. Meanwhile, LXA4 receptor antagonist Boc-2 reversed the protective effects of LXA4 on intestinal injury but failed to affect the oxidative stress and the related Nrf2 pathway. Furthermore, Nrf2 antagonist brusatol reversed the antioxidant effects conferred by LXA4 and led to exacerbation of intestinal epithelium cells oxidative stress and apoptosis, finally resulting in a decrease of survival rate of rat. Meanwhile, LXA4 pretreatment upregulated nuclear Nrf2 level and reduced hypoxia/reoxygenation-induced IEC-6 cell damage and Nrf2 siRNA reversed this protective effect of LXA4* in vitro*. In conclusion, these findings suggest that LXA4 ameliorates IIR injury by activating Keap1/Nrf2 pathway in a LXA4 receptor independent manner.

## 1. Introduction

Intestinal ischemia reperfusion (IIR), a critical condition usually caused by many clinical scenarios such as shock, acute mesenteric ischemia, sepsis, mesenteric thrombosis, or bowel transplantation [1], is closely related to high morbidity and mortality [2]. Interruption of blood supply to intestine could result in tissue damage. However, the subsequent blood flow restoration may lead to additional injury, referred to as reperfusion injury [3]. During reperfusion, when oxygen is supplied in large amounts to the tissues that is deprived of oxygen, the electrons from xanthine oxidase are transferred to molecular oxygen, forming large quantities of superoxide radicals [4]. Thus, far greater amounts of oxidants are produced after restoration of blood supply to the ischemic tissues. This causes damage to the intestinal mucosa and impairment of the local microvasculature, leading to high permeability of vascular and mucosa breakdown and subsequent systemic sepsis and multiple organ failure [5], which contributes to a high mortality in the critical care setting. Currently, effective preventive therapy of IIR injury is lacking.

Due to the great involvement of reactive oxygen species (ROS) in the pathophysiology of IIR injury, antioxidants have been employed by researchers in an attempt to alleviate the injury caused by IIR [6–8]. Nrf2 is a stress sensing genetic transcription factor that functions as a master regulator of cellular responses to oxidative damage and other stressful conditions [9]. The Nrf2 antioxidant response pathway is “the primary cellular defense against the cytotoxic effects of oxidative stress” [10]. Recently, our studies showed that the Nrf2 pathway took part in intestine ischemia reperfusion injury and that enhancing Nrf2 nuclear translocation could effectively attenuate postischemic intestinal mucosal impairment [11]. It, therefore, suggests that increasing endogenous antioxidative capacity would confer protective effects in conditions that may cause IIR injury.

**Figure 1:**
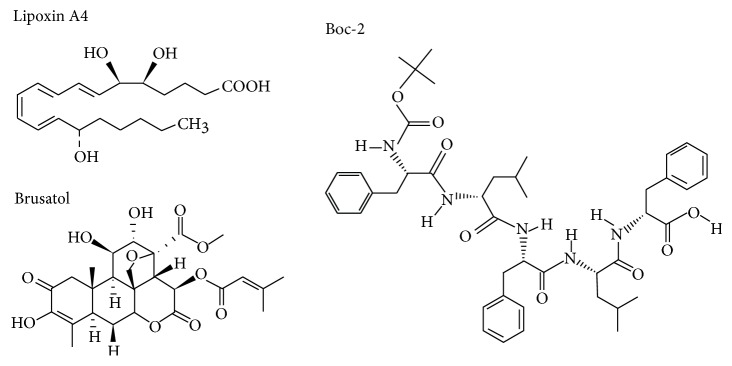
Chemical structure of Lipoxin A4 (C_20_H_32_O_5_), Boc-2 (C_44_H_59_N_5_O_8_), and brusatol (C_26_H_32_O_11_).

Lipoxins (LXs) are members of the family of bioactive products generated from arachidonic acid [12]. These molecules used to be identified* in vivo* as the first endogenous “braking signals” of inflammation [13]. Of note, recent studies showed that LXs also played an important role in the prevention of oxidative stress-mediated tissue injury [14–17]. Lipoxin A4 (LXA4) (C_20_H_32_O_5_, Figure 1) is one of the most important LXs and can bind to the LXA4 receptor (ALXR), a specific G-protein-coupled receptor [18]. In addition, LXA4 may exert biological effects through other mechanisms where the protective effect of LXA4 was found to be partially mediated by transcription factor peroxisome proliferator-activated receptors gamma (PPAR*γ*) in experimental brain ischemia reperfusion in rodents [19]. However, few reports concern its function on antioxidative stress and its impact on endogenous antioxidant signaling pathway during IIR* in vivo*.

We hypothesized that the antioxidant effects of Lipoxin A4 are mediated via Nrf2 pathway activation, which may effectively attenuate IIR injury in the early stage. The current study was aimed to investigate whether Lipoxin A4 preconditioning could mitigate intestinal mucosa injury and increase postischemic survival rate through reducing oxidative stress via activating Keap1/Nrf2 pathway in a rat model of IIR injury.

## 2. Materials and Methods

### 2.1. Animals

Male Sprague-Dawley rats (weighing 200–250 g) were housed in individual cages in a temperature-controlled room with 12-h light-dark cycles in the Department of Laboratory Animal Center at Sun Yat-sen University with a specific pathogen-free (SPF) grade, laminar flow atmosphere. All animals were fasted overnight before the experiment but had free access to water. Animal study protocols were approved by the Sun Yat-sen University Animal Care Committee, and the experiments were performed in adherence to the guidelines provided by the National Institutes of Health for the use of animals in laboratory experiments.

### 2.2. Intestine Ischemia and Reperfusion

Rats were anesthetized with pentobarbital (30 mg/kg body weight) via intraperitoneal injection under 50% oxygen delivered using an animal mask as the previous study [20]. The intestinal ischemia was established by occluding the superior mesenteric artery (SMA) and confirmed by immediate blanching of the small intestine and cecum. After ischemia for 45 minutes (min), vascular clamp was removed and SMA was reflowed, which was recognized by returning to gut's original color [21]. During the experiments, temperature of animals was maintained in normothermia (37°C) with heating pads. After reperfusion for 6 hours, rats were sacrificed subsequent to being deeply anesthetized with chloral hydrate (400 mg/kg body weight, i.p.). The sham group was subjected to the surgical procedures described below without intestine and vessel manipulation and maintained under anesthesia for the same time as the IIR group in terms of duration of the experiment.

### 2.3. Treatment Protocols

Forty-eight SD rats were randomly divided into eight groups (*n* = 6 per group): sham, IIR model, sham+Lipoxin A4 (LXA4), IIR+LXA4, sham+LXA4+Boc-2 (Boc-2, C_44_H_59_N_5_O_8_, a LXA4 antagonist, Figure 1), IIR+LXA4+Boc-2, sham+LXA4+brusatol (Bru., C_26_H_32_O_11_, a Nrf2 antagonist, Figure 1), and IIR+LXA4+Bru.

First, to evaluate the effects of Lipoxin A4 on IIR injury, Lipoxin A4 (100 *μ*g/kg [22]) (Cayman Chemical Company, Ann Arbor, USA) was dissolved in normal saline 2 mL/kg and administrated via tail vein 15 min before intestine ischemia in IIR+LXA4, IIR+LXA4+Boc-2, and IIR+LXA4+Bru groups, and the same dose of Lipoxin A4 was also given in sham+LXA4 as a control. Next, to detect the role of Lipoxin A4 receptor in the protective effects of Lipoxin A4, Lipoxin A4 receptor antagonist Boc-2 (50 mg/kg [23], i.p., Phoenix Pharmaceuticals, Phoenix, AZ) was given 10 min before Lipoxin A4 administration in IIR+Boc-2 and IIR+LXA4+Boc-2 groups. Boc-2 was also given in sham+LXA4+Boc-2 as a control. Finally, to expose the role of Nrf2 pathway in Lipoxin A4-conferred intestinal mucosa protection, Nrf2 antagonist brusatol (0.4 mg/kg [24], i.p., BOC Sciences, Shirley, NY, USA) was administrated every other day for a total of five times before surgery in IIR+LXA4+Bru group. Brusatol was also given in sham+LXA4+Bru as a control.

### 2.4. Intestinal Mucosal Injury Scoring

Intestine paraffin section hematoxylin-eosin staining was used for scoring. Injury degree of intestinal mucosa was initially evaluated independently by two pathologists who were blinded to the study groups. Using a modified Chiu's method [25], degree of intestine injury was evaluated according to changes of the glands and villus of the intestinal mucosa. A minimum of five randomly chosen fields in intestine H&E staining section from each rat were evaluated and averaged to evaluate mucosal damage degree.

### 2.5. Sample Collection

Blood samples were collected from abdominal aortography and centrifuged at 3,500 revolutions/min (rpm) for 15 min at 4°C. The plasma was used for subsequent measurements. A segment of 1 cm intestine was cut 5 cm away from the terminal ileum for paraffin embedding. Another segment of 4 cm was cut and washed with cold saline, and intestinal mucosa was scraped off, dried with suction paper, and preserved at −80°C for further detection.

### 2.6. Cell Culture and Hypoxia/Reoxygenation (H/R) Protocol

IEC-6 cells were obtained from American Type Culture Collection (ATCC, Manassas, VA, USA) and cultured in Dulbecco's Modified Eagle's Medium supplemented with 10% fetal bovine serum and 1% penicillin/streptomycin antibiotics. Cells were grown at 37°C in an atmosphere of 5% CO_2_ in air. The H/R protocol was performed as previously described with modifications [26]. Briefly, for hypoxia, confluent cells were incubated in an anaerobic chamber equilibrated with 5% CO_2_ and 95% N_2_. A metal catalyzer (Engelhard, USA) was used to maintain a constant low oxygen concentration (<0.1%) in the chamber. After a 12-hour hypoxia period, cells were transferred back to a regular incubator with 21% oxygen for 6 hours. Control experiments were performed by omitting hypoxia and incubating the cells for 18 hours under normoxic conditions.

### 2.7. Nrf2 siRNA Transfection and Lipoxin A4 Delivery in IEC-6 Cells

IEC-6 cells were maintained in Dulbecco's Modified Eagle's Medium supplemented with 10% fetal bovine serum and 1% penicillin/streptomycin antibiotics (5% CO_2_) at 37°C. Commercial Nrf2 siRNA (Santa Cruz Biotechnology) was used for inhibition of Nrf2 expression according to the manufacturer's protocol. After transfection with nonsilencing control siRNA and Nrf2 siRNA, cells were incubated in Dulbecco's Modified Eagle's Medium for 48 hours. Each experiment was performed at least in triplicate. IEC-6 cells were collected and mRNA expression of Nrf2 level was detected by real time Polymerase Chain Reaction using the following primers: 5′-GGTGATGAATTTTACTCTGC-3′ (sense) and 5′-TTTCCGAGTCACTGATGAACC-3′ (anti-sense) for Nrf2 and 5′-GTCGGTGTGAACGGATTT-3′ (sense) and 5′-ACTCCACGACGTACTCAGC-3′ (anti-sense) for GAPDH. Before H/R injury, the cells in some of the subgroups were exposed to Lipoxin A4 (10 nM) for 12 hours [27].

### 2.8. Enzyme-Linked Immunosorbent Assay

Diamine oxidase (DAO) (Cloud-Clone Crop, USA), D-lactic acid (D-LA) (Biosamite, USA), and intestinal-type fatty acid-binding protein (FABP2) (Cloud-Clone Crop, USA) in serum were measured according to the manufacturer's instructions of enzyme-linked immunosorbent assay (ELISA) kits as previously described [20].

### 2.9. Determination of 15-F2t-Isoprostane Content and Superoxide Dismutase Activity

15-F2t-isoprostane, a specific marker of oxidative stress, was measured using an enzyme-linked immunoassay kit as we described [28]. Homogenized intestinal mucosa tissues were purified using Affinity Sorbent and Affinity Column (Cayman Chemical, Ann Arbor, MI) and then processed for analysis. The values of free 15-F2t- isoprostane were expressed as pg/g wet protein in tissue homogenates. The activity of superoxide dismutase (SOD) was determined using a commercial kit (Nanjing Jiancheng Bioengineering Institute, Nanjing, China) as we described. SOD activity was expressed as unit/mg wet protein.

### 2.10. Lactate Dehydrogenase (LDH) Assay

IEC-6 cells were seeded at low density (10,000 cells/cm^2^) in 96-well plates. After experimental treatment, LDH assays, as measured for cell injury degree, were carried out according to the manufacturer's introduction (Roche Diagnostics, Indianapolis, USA) as described in our previous study [28].

### 2.11. Immunofluorescence

For immunofluorescence, the intestine paraffin blocks were cut into 5 *μ*m sections; potential nonspecific staining in the sections was blocked with 5% bovine serum albumin and 0.3% Triton X-100 in PBS. Mouse anticytochrome C (1 : 200) (Santa Cruz, California, USA) antibody was used as primary antibodies and then followed by a secondary antibody conjugated with fluorescence (1 : 100) (Life Technologies, USA). Fluorescent microscope (Leica, DMLB2, Germany) was utilized for viewing the stained sections. Five randomly selected fields of each slide were semiquantified and averaged using the software Image J 1.48 (National Institutes of Health) according to its instructions.

### 2.12. TUNEL Staining

Apoptosis in the intestine sections was examined after Terminal Deoxynucleotidyl Transferase dUTP Nick-End Labeling (TUNEL) staining with* in situ* cell death detection kit (Roche, Basel, Switzerland) as previously described [29]. The hematoxylin (Sigma-Aldrich, St. Louis, MO) was used to stain nuclei. The average number of apoptotic positive cells was calculated from five random fields.

### 2.13. Western Blot Assay

Rat intestinal mucosa homogenate or cell suspension was resolved by 8–10% sodium dodecyl sulfate polyacrylamide gel electrophoresis and transferred to nitrocellulose membranes and processed as described [30]. Monoclonal antibody to Nrf2 antibodies (1 : 200, sc-722, Santa Cruz, California, USA), Keap1 antibodies (1 : 500, ABS97, Millipore), and HO-1 (1 : 200, sc-10789, Santa Cruz, California, USA) were used as primary antibodies, and the HP-conjugated anti-mouse IgG (Cell Signaling Technology) as secondary antibody. Protein bands were detected by ECL kit (enhanced chemiluminescence detection KGP1125, Nanjing KeyGen Biotech. Co., Ltd.). Anti-histone H2A (1 : 1000, Santa Cruz, California, USA) and anti-GAPDH (1 : 2000, Merck Millipore, Germany) were used as loaded sample reference to normalize relative level of each detected protein.

### 2.14. Survival Rates

Survival rates were assayed in another 4 groups (sham, IIR, IIR+LXA4, and IIR+LXA4+Bru, *n* = 18 per group). The survival rates of each group were observed for duration of 72 hours from the onset of intestine reperfusion in IIR model.

### 2.15. Statistical Analysis

Biochemical assays were performed in triplicate for each specific sample. Therefore, all the data points are means of numbers that themselves are means of triplicate measurements for these parameters. Data are expressed as mean ± standard error of the mean. Significance was evaluated using* one-way ANOVA* test (SPSS 13.0, SPSS Inc., Chicago, III) followed by* Tukey post hoc* multiple comparisons test for unpaired values. *p* < 0.05 was considered statistically significant.

## 3. Results

### 3.1. Lipoxin A4 Attenuated Intestinal Mucosa Damage after IIR Injury

**Figure 2:**
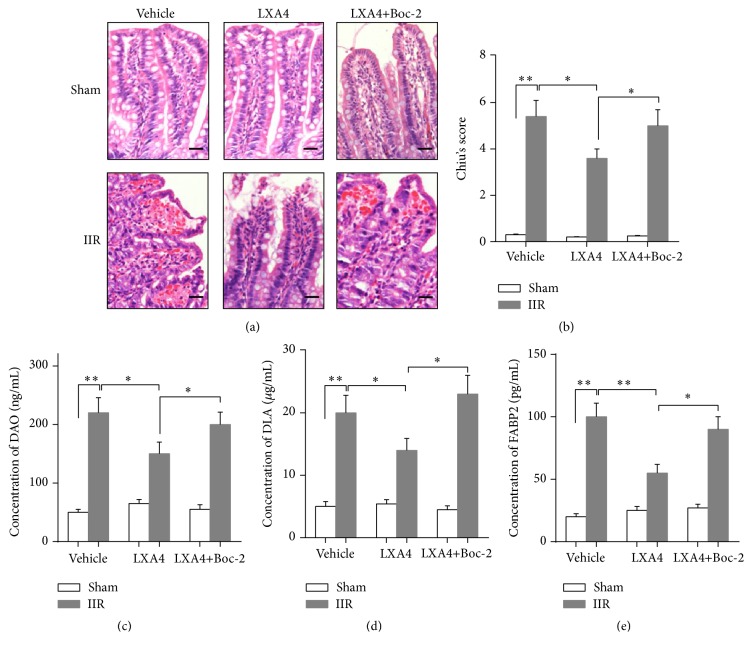
Effects of Lipoxin A4 on intestine ischemia reperfusion (IIR) injury. Representative photomicrographs (400x) showing H&E staining of intestine (a) and Chiu's score (b) was carried out to evaluate the injury degree; quantitative analysis using ELISA method was taken to assay the concentration of diamine oxidase (DAO) (c), D-lactic acid (DLA) (d), and intestinal-type fatty acid-binding protein (FABP2) (e) in serum. Each bar represents the mean ± SEM (*n* = 6 per group). ^*∗*^ *p* < 0.05, ^*∗∗*^ *p* < 0.01,* one-way ANOVA* with* Tukey test*.

As shown in Figure 2(a), normal villi were observed in sham group, but after 45 min of intestine ischemia and 6 hours (h) of reperfusion, the intestinal mucosa was impaired, manifested as destruction ranged from extended subepithelial with necrotic epithelial cell sheets at the tips of the villi to denuded villi with lamina propria and dilated capillaries exposed. Compared with sham group, modified Chiu's score (Figure 2(b)) was significantly increased in IIR group, accompanied with elevation of serums DAO, DLA, and FABP2 levels (*p* < 0.05, Figures 2(c)–2(e)). However, Lipoxin A4 preconditioning significantly reduced intestinal mucosa injury induced by IIR, evidenced as lower Chiu's score and serums DAO, DLA, and FABP2 levels than those in IIR groups (*p* < 0.05). Furthermore, Lipoxin A4 receptor (ALXR) antagonist Boc-2 partly reversed the protective effects of Lipoxin A4, evidenced as worsening pathological change and increase of Chiu's score and serums DAO, DLA, and FABP2 levels. These results indicated that Lipoxin A4 preconditioning conferred protective effects against IIR injury, which involved the activation of ALXR.

### 3.2. Keap1/Nrf2 Pathway Was Activated by Lipoxin A4 Preconditioning in an ALXR Independent Manner

**Figure 3:**
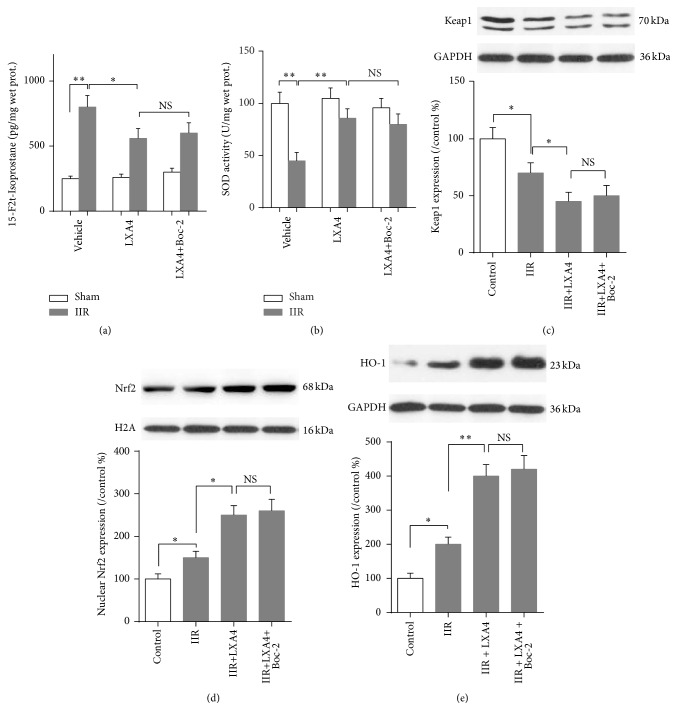
Effects of Lipoxin A4 on Keap1/Nrf2 pathway. Quantitative analysis using ELISA method was taken to assay the concentration of oxidative marker 15-F2t-Isoprostane (a) and SOD activity (b) in intestine mucosa. Representative Western blots and quantitative analyses showing total Keap1 (c) and HO-1 (d) protein and nuclear Nrf2 (e) protein expressions in intestine mucosa. Each bar represents the mean ± SEM (*n* = 6 per group). ^*∗*^ *p* < 0.05, ^*∗∗*^ *p* < 0.01,* one-way ANOVA* with* Tukey test*. NS means no significant difference.

To further explore the underlying mechanism of Lipoxin A4 preconditioning, intestinal mucosa oxidative stress level was detected. As shown in Figures 3(a) and 3(b), after 45 min of intestine ischemia and 6 h of reperfusion, oxidative stress was significantly increased in intestinal mucosa, manifested as increase of oxidative marker 15-F2t-Isoprostane and decrease of SOD activity (*p* < 0.05 versus group sham). Moreover, Lipoxin A4 preconditioning reduced mucosa 15-F2t-Isoprostane release and increased SOD activity with concomitant elevation of nuclear Nrf2 and total HO-1 protein expression and reduction of Keap1 protein expression (Figures 3(c)–3(e)). However, after blocking the ALXR by Boc-2, no change was exhibited in Keap1/Nrf2 pathway and the associated 15-F2t-Isoprostane and SOD activity (*p* > 0.05 versus IIR+LXA4). These results suggested that the protective effects conferred by Lipoxin A4 preconditioning were associated with Keap1/Nrf2 pathway activation, which could be activated independent of ALXR.

### 3.3. Nrf2 Inhibition Reversed the Intestinal Mucosa Protective Effects Conferred by Lipoxin A4 Preconditioning

**Figure 4:**
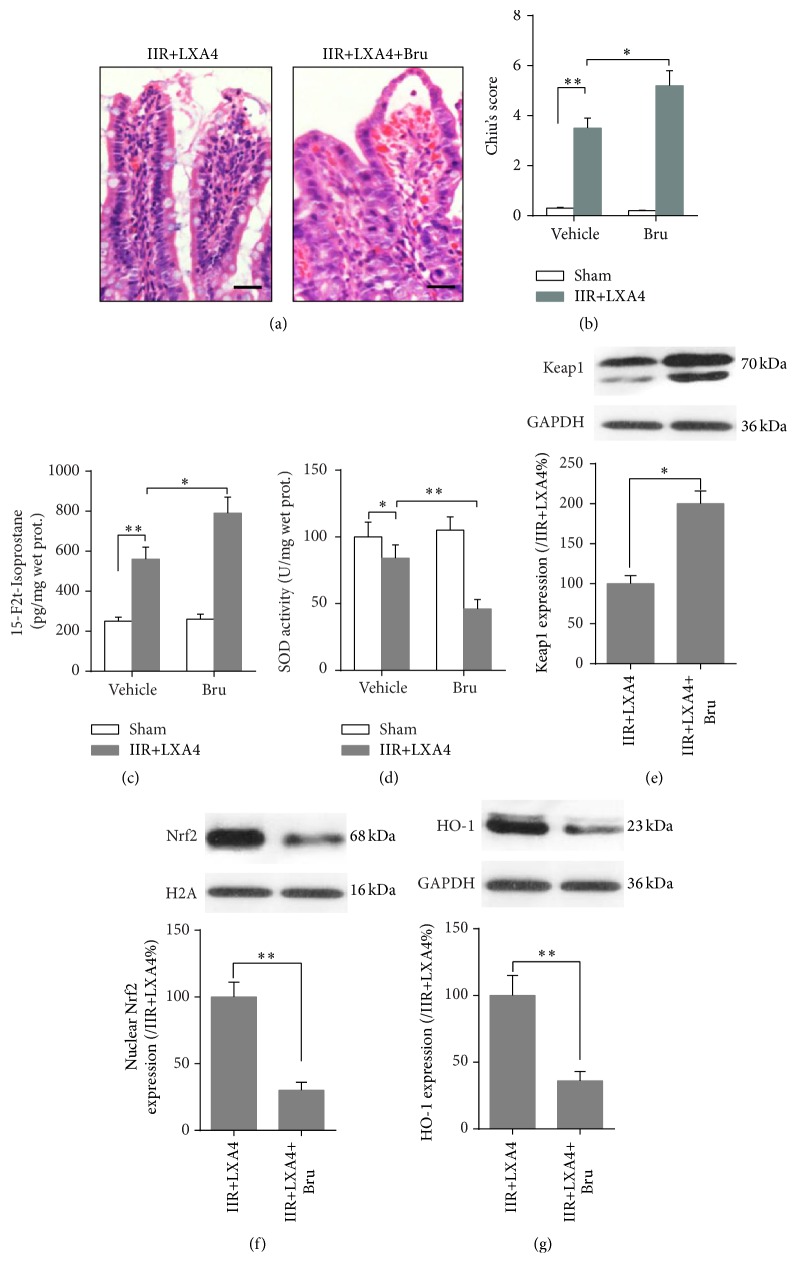
Brusatol reversed the protective effects conferred by Lipoxin A4. Representative photomicrographs (400x) showing H&E staining of intestine (a) and Chiu's score (b) was carried out to evaluate the injury degree; quantitative analysis using ELISA method was taken to assay the concentration of oxidative marker 15-F2t-Isoprostane (c) and SOD activity (d) in intestine mucosa. Representative Western blots and quantitative analyses showing total Keap1 (e) and HO-1 (g) protein and nuclear Nrf2 (f) protein expressions in intestine mucosa. Each bar represents the mean ± SEM (*n* = 6 per group). ^*∗*^ *p* < 0.05, ^*∗∗*^ *p* < 0.01,* one-way ANOVA* with* Tukey test*.

Next, Nrf2 inhibitor brusatol was employed to determine the relationship between Lipoxin A4 preconditioning and Keap1/Nrf2 pathway. As shown in Figure 4, after inhibiting Nrf2 with brusatol before Lipoxin A4 intervention, nuclear Nrf2 protein expression was significantly decreased, along with decrease of HO-1 protein expression and increase of Keap1 protein expression, leading to an elevation of oxidative stress, manifested by increase of oxidative marker 15-F2t-Isoprostane and decrease of SOD activity, and finally resulting in higher Chiu's score and worsening pathologic morphology (*p* < 0.05 versus IIR+LXA4). These results indicated that Lipoxin A4 preconditioning attenuated intestinal mucosa damage after IIR injury via activating Keap1/Nrf2 pathway and the subsequent increase of downstream antioxidative enzyme HO-1 protein expression.

### 3.4. Lipoxin A4 Preconditioning Attenuated Intestinal Epithelium Cells Apoptosis and Increased Rat Survival Rate via Keap1/Nrf2 Pathway Activation

**Figure 5:**
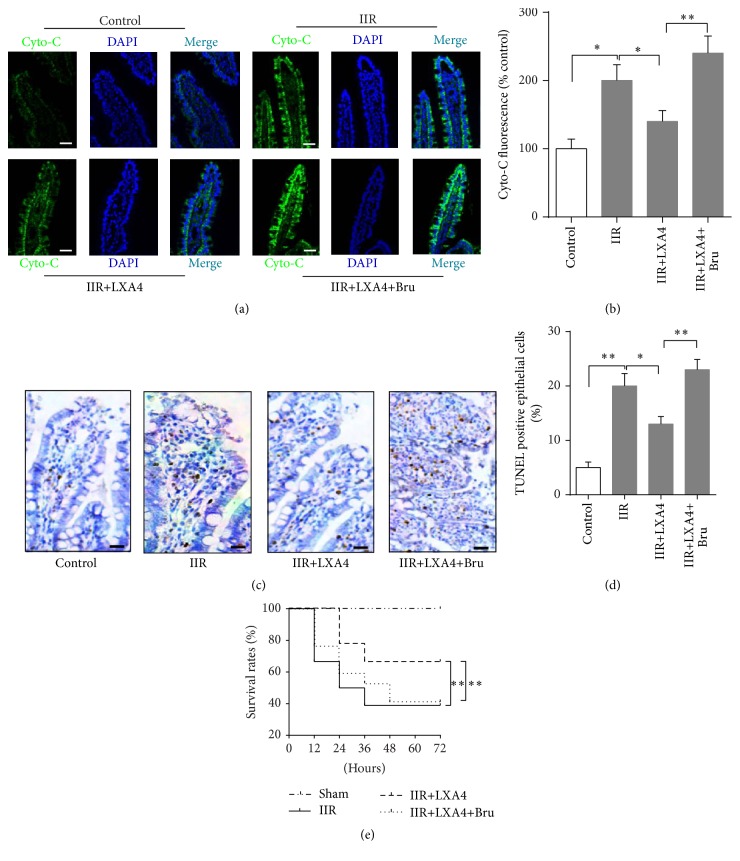
Lipoxin A4 attenuated intestinal epithelium cells apoptosis and finally increased rat survival rate. Representative photomicrographs showing fluorescent staining (a, 400x) of cytochrome C (green) or DAPI (blue) in intestinal epithelium cells and fluorescence intensity were measured (b). Cell apoptosis was also measured by using TUNEL assay (c, 400x) and TUNEL positive cells were counted (d). Each bar represents the mean ± SEM (*n* = 6 per group); the survival rate (e) was determined during 72 hours after reperfusion; *n* = 18 per group. ^*∗*^ *p* < 0.05, ^*∗∗*^ *p* < 0.01,* one-way ANOVA* with* Tukey test*.

As shown in Figure 5, IIR significantly increased the fluorescence intensity of cytochrome C (Figures 5(a) and 5(b)) and TUNEL (Figures 5(c) and 5(d)) positive cells in intestinal mucosa at 6 hours after reperfusion, which was related to a low rate of survival (40%) during 72 hours after reperfusion. In contrast, Lipoxin A4 preconditioning could effectively reduce the fluorescence intensity of cytochrome C and TUNEL positive cells in intestinal mucosa and significantly increased rats' survival rate (68%, *p* < 0.05 versus IIR). However, Nrf2 antagonist brusatol given before Lipoxin A4 preconditioning completely reversed the protective effects conferred by Lipoxin A4, evidenced as higher fluorescence intensity of cytochrome C and TUNEL positive cells in intestinal mucosa than those in IIR+LXA4 group (*p* < 0.05), associated with decrease of survival rate (42%, *p* < 0.05 versus IIR+LXA4). These results indicated that Lipoxin A4 preconditioning could elevate rats' survival rate via alleviating intestinal epithelium cells apoptosis in a Keap1/Nrf2 pathway dependent manner in rat IIR model.

### 3.5. Knockdown of Nrf2 Reversed the Protection Effects of Lipoxin A4 on Preventing IEC-6 Cells from H/R Injury

**Figure 6:**
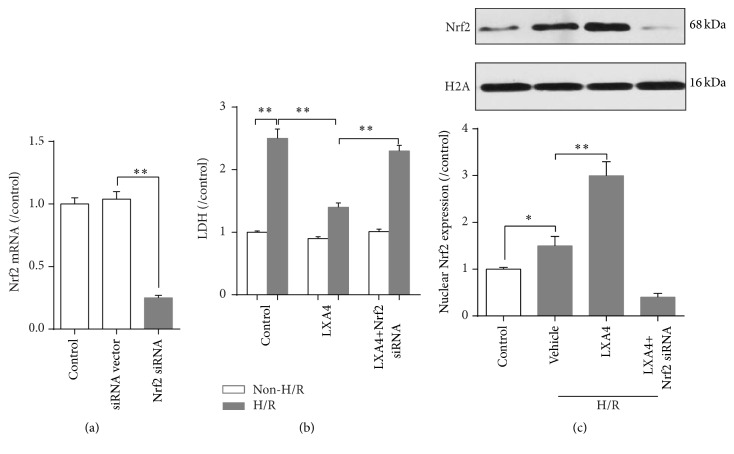
Effects of Lipoxin A4 and knockdown of Nrf2 on H/R-induced cell damage in IEC-6 intestinal epithelium cells. (a) Effects of gene knockdown by Nrf2 siRNA. (b) Relative lactate dehydrogenase (LDH) release of IEC-6 cells. (c) Determining nuclear Nrf2 expression by western blot. Bars are mean ± standard deviation from four independent experiments. ^*∗*^ *p* < 0.05, ^*∗∗*^ *p* < 0.01,* one-way ANOVA* with* Tukey test*.

To confirm the effects of Lipoxin A4 on intestinal epithelium cells, an IEC-6 cells H/R model was used in our study. First, in IEC-6 cell line, the gene knockdown effect of Nrf2 was determined and the PCR result has shown that, after being treated with Nrf2 siRNA for 12 hours, the mRNA of Nrf2 was significantly decreased (*p* < 0.01 versus siRNA vector; Figure 6(a)). Then, we confirmed that effects of Lipoxin A4 pretreatment on IEC-6 cells exposed to H/R. Lipoxin A4 (10 nM) could dramatically decrease LDH release from IEC-6 cells compared with the control group (*p* < 0.01,Figure 6(b)) and elevate the Nrf2 protein expression (Figure 6(c)) which was decreased after H/R injury. However, Nrf2 siRNA significantly reversed this protective effect, evidenced by increased LDH release.

## 4. Discussion

Aberrant synthesis of reactive oxygen species and accumulation of oxidative products within the intestinal mucosa are believed to be a chief culprit for the pathogenesis of intestine injury caused by IIR [31]. Increased levels of Nrf2 nuclear translocation are associated with repair of injured intestinal mucosa in animal models [32]. Here, the current study addresses the question of whether the antioxidant effects of Lipoxin A4 were mediated via Nrf2 pathway activation in rat ischemia reperfusion injury model. There are several major findings in the current study. Firstly, Lipoxin A4 conferred an antioxidative effect against intestinal mucosa injury following IIR and protected IEC-6 cells from H/R injury, which was mediated by enhancing endogenous Keap1/Nrf2 signaling. Furthermore, the effect of Lipoxin A4 was developed in a Lipoxin A4 receptor independent manner.

LXA4 was identified as a short lived lipid and described as a potent endogenous bioactive anti-inflammatory lipid mediator while its antioxidant effects have been recently reported [14]. LXA4 may exert biological effects through binding to ALXR. In the present study, the protective effect of LXA4 on IIR-induced intestinal mucosa injury is in principle in line with several other studies which demonstrated protective effects for pharmacologically delivered Lipoxins or stable Lipoxin analogues in pathologies with I/R [33–35], and these protective effects can be reversed by Boc-2, a specific antagonist of ALXR [36]. Therefore, the protective effect of LXA4 on intestinal mucosa seen in the current study may be partly related to ALXR activation, consisted with results of Tang et al. which showed that activating ALXR with BML-111 could attenuate lung injury in endotoxemic mice and mitigate endothelial hyperpermeability, while Boc-2 reversed this effect [37]. However, Wu et al. found that the effect of LXA4 on oxygen-glucose deprivation (OGD)/recovery- (OGDR-) induced astrocyte injury cannot be reversed by Boc-2, and the effect of LXA4 on astrocytic cell damage may be not related to ALXR [38]. The difference of these results may be related to the special pathophysiology of the disease and the different underlying mechanisms remain to be explored.

We recently showed that NF-E2-related factor-2 (Nrf2) played an important role in IIR injury through regulating a major environmental and oxidative stress response [11]. Nrf2 is held in the cytoplasm by a cytoskeletal-associated specific inhibitory protein, the Kelch-like ECH associating protein 1 (Keap1) in normal quiescent cells. Upon stimulation by oxidative stress, cysteine residues within the hinge region of Keap1 can be modified and cause a conformational change, resulting in the loss of Nrf2 binding [39]. The Nrf2 is then translocated into the nucleus and binds to the promoter region of a number of genes encoding antioxidative and phase 2 enzymes, such as superoxide dismutase (SOD) and heme-oxygenase 1 (HO-1), which can antagonize oxidative stress induced by reactive oxygen species [40]. In our current study, we found that Lipoxin A4 preconditioning activated the transcription factor Nrf2 and subsequently increased the expression of HO-1, leading to reduction of apoptosis of intestinal epithelium cells. Similarly, Lipoxin A4 has also been found to exert antioxidative effects to ameliorate ischemic brain injury by directly activating another transcription factor PPAR-*γ* [19]. In addition, Lipoxin A4 could suppress lipopolysaccharide-induced HeLa cell proliferation and migration via NF-*κ*B pathway [41]. These suggest that Lipoxin A4 could activate multiple signal transduction pathways to confer protective effects in a variety of I/R disorders and that Lipoxin A4 preconditioning may be a prospective intervention in reducing intestinal mucosa injury induced by IIR. Even more interesting is that we found Lipoxin A4 activated Keap1/Nrf2 pathway in an ALXR independent manner, indicating that the protective effects of Lipoxin A4 in IIR injury may be mediated by both receptor-binding and Nrf2 nuclear translocation pathways, which could function independently.

Of note, Lipoxin A4 postconditioning also has been reported to provide protective effects on myocardial ischemia/reperfusion injury [42], but whether or not it can protect against intestinal mucosa injury induced by IIR needs to be further investigation. Meanwhile, blocking Lipoxin A4 receptor (ALXR) using Boc-2 could aggregate intestinal mucosa injury with concomitant increase in serums DAO, DLA, and FABP2. This suggests that activating ALXR represents a mechanism whereby Lipoxin A4 preconditioning confers protection against IIR injury. However, the downstream signaling following Lipoxin A4 preconditioning mediated binding to ALXR with subsequent protective effects on IIR requires future study although there was study indicating that it may be through p38 MAPK pathway activation [43]. Moreover, Lipoxin A4 pretreatment has been shown as an effective method for preventing ischemia/reperfusion-caused intestine injury in our current study. However, it is difficult to predict the onset of ischemia in some clinical settings that may cause IIR. In the further study, Lipoxin A4 treatment will be employed at the time of reperfusion instead of being given before ischemia, because the implementation of intestinal protective therapy at the time of reperfusion could be clinically feasible as it is more predictable and is under the clinician's control.

**Figure 7:**
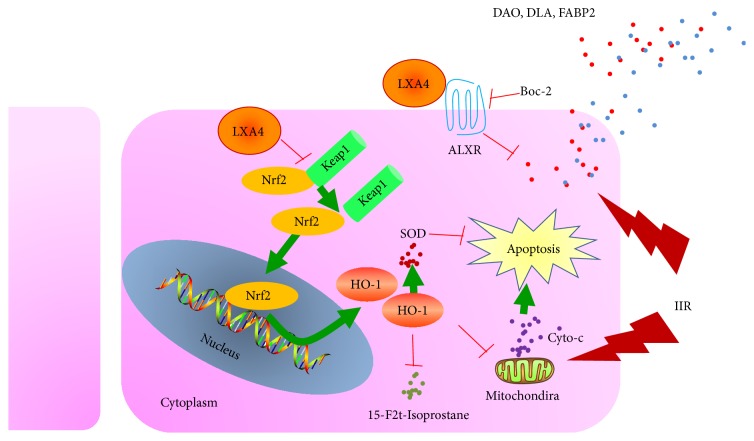
Proposed signaling mechanisms mediated by Lipoxin A4 in preventing intestinal epithelium cells from intestine ischemia reperfusion (IIR) injury. Lipoxin A4 promotes Keap1 dissociation from Nrf2 and leads to Nrf2 translocation from cytoplasm to nucleus, resulting in enhancing downstream HO-1 gene expression without binding to the Lipoxin A4 receptor (ALXR). Antioxidant enzyme HO-1 could possibly reduce cytochrome C release from mitochondria and decrease lipid peroxidative product 15-F2t-Isoprostane and elevate cell antioxidant ability evidenced by increase of superoxide dismutase (SOD) and finally decrease cells apoptosis. In addition, Lipoxin A4 binding to ALXR could also reduce damage of diamine oxidase (DAO) products, D-lactic acid (DLA), and intestinal-type fatty acid-binding protein (FABP2) release with other mechanisms.

In summary, we have discovered that Nrf2 is involved to serve as an antioxidant enzyme gene transcription factor in combating intestinal mucosa injury induced by IIR. Lipoxin A4 preconditioning could effectively attenuate intestinal mucosa oxidative stress via activating Keap1/Nrf2 pathway and subsequently enhance downstream antioxidant enzyme HO-1 production, highlighting the importance of enhancing Nrf2 nuclear translocation in IIR injury prevention. Lipoxin A4 receptor inhibition, however, seems to have no effects on Keap1/Nrf2 pathway. The data presented in the current study support the notion that Lipoxin A4 preconditioning attenuated intestine injury via activation of Keap1/Nrf2 in a receptor independent way (Figure 7). Therefore, enhancing the efficiency of Nrf2 nuclear translocation may provide a novel approach for promoting the protective effects of Lipoxin A4 intervention.
